# Accelerating Saturate, Aromatic, Resin, Asphaltene (SARA) Analysis for High-Fidelity Petroleum Profiling via μSARA-HPLC

**DOI:** 10.1021/acsomega.5c00439

**Published:** 2025-06-23

**Authors:** Ibrahim Atwah, Maram Alsaif, Muhammad Usman, Mohammed Abualreesh

**Affiliations:** † EXPEC Advanced Research Center, 36516 Saudi Aramco, P.O. Box 5000, Dhahran 31311, Saudi Arabia; ‡ Physical Science and Engineering Division, 127355 King Abdullah University of Science and Technology (KAUST), P.O. Box 4700, Thuwal 23955-6900, Saudi Arabia; § Jackson School of Geosciences, The University of Texas at Austin, 2305 Speedway Stop C1160, Austin, Texas 78712-1692, United States

## Abstract

Saturates, aromatics, resins, and asphaltenes (SARA) analysis is essential for petroleum characterization, providing critical insights into crude oil properties that impact exploration, production, refining, and transport. However, many current methods have issues such as long runtime, system complexity, and reproducibility challenges. To address these issues, this study introduces μSARA-HPLC, an optimized, automated SARA analysis system using high-performance liquid chromatography (HPLC) with fraction collection. The system features three columns: a Polaris Si-SA MetaGuard column for asphaltene precipitation, a ZORBAX SB-CN column for resin separation, and a ZORBAX RX-SIL column for aromatics. The solvent gradients are carefully controlled, with mid-run flow reversal to ensure comprehensive separation. Elution windows are set using model compounds and verified via gas chromatography–mass spectrometry (GC–MS) for saturates and aromatics and Fourier transform ion cyclotron resonance mass spectrometry (FT–ICR–MS) for resins and asphaltenes. The system runs at 1.5 mL/min with *n*-pentane, dichloromethane, methanol, and isopropanol, with each run completed in 35 min, reducing the analysis time significantly compared with existing methods. Sample use is minimized and column life is extended by using injection volumes of 1.5–3 μL from 1:100 dilutions of oil (10 mg of oil in 100 μL of DCM). Dual detection using an evaporative light scattering detector (ELSD) and a diode array detector (DAD) provides exceptional sensitivity for all fractions, achieving detection limits as low as 0.05% by weight and an average recovery rate of 99.52%. Reproducibility was validated across crude oils and bitumen with varying API gravities, including extra-heavy and ultralight samples, using the NSO-1 reference oil over four months, which resulted in less than 1% deviation. This novel system provides a fast, reliable, and scalable approach to SARA analysis, advancing standardization in research and industry.

## Graphical abstract

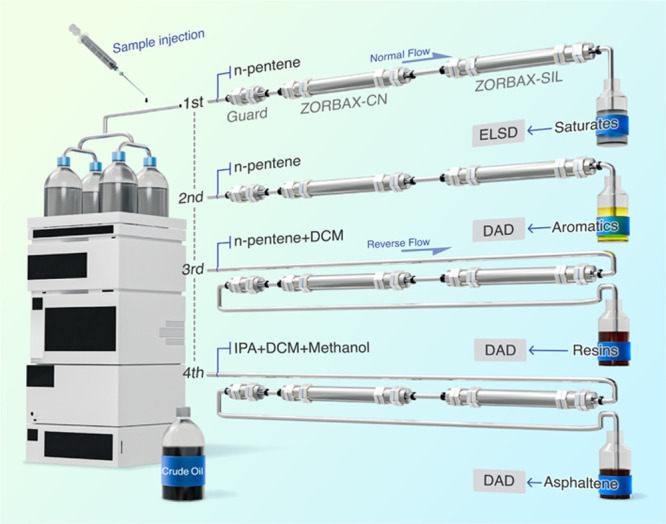

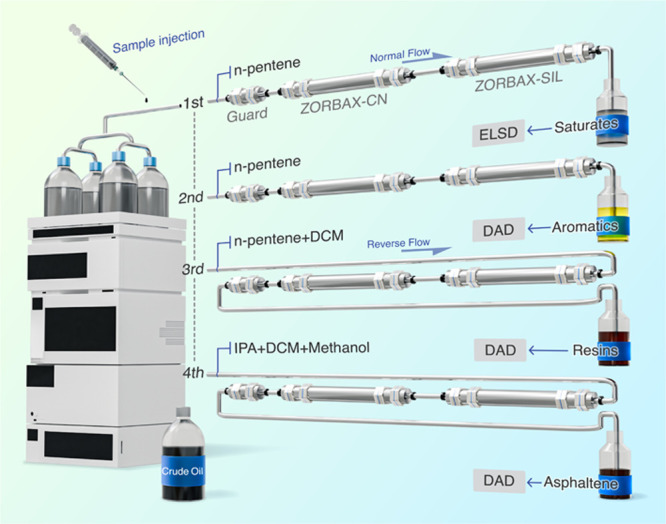

## Introduction

Petroleum forms through the thermal maturation of organic matter, primarily kerogen, that has accumulated in sedimentary basins over millions of years.^1^ Under the influence of heat and time, this organic material transforms within source rocks into hydrocarbons. The complexity of kerogen, shaped by its biological origins and the geological history of the basin, varies widely, as seen in source rocks from periods such as the Devonian.^2,3^ As petroleum matures, it becomes a complex mixture of hydrocarbons that challenges geochemists in their efforts to characterize these fluids. Early approaches to understanding petroleum composition relied on the solubility of petroleum compounds in organic solvents; the compounds were divided into maltenes—components that are soluble—and asphaltenes, which are insoluble. The maltenes were further separated into three fractions: saturates, aromatics, and resins.^4,5^ Together with asphaltenes, these four fractions form the basis of SARA analysis, a key method used to categorize the major components of petroleum and better understand its chemical behavior.

In SARA group-type characterization, crude oil is classified into four distinct fractions on the basis of polarity and solubility: saturates, aromatics, resins, and asphaltenes. The saturated fraction consists of nonpolar hydrocarbons, which typically exist as linear, branched, or cyclic molecules, including *n*-alkanes, iso-alkanes, and cycloalkanes (Figure 1). These fully saturated hydrocarbons, composed solely of carbon and hydrogen with no significant heteroatom content, are considered the most valuable component of crude oil due to their low polarity. In contrast, aromatics contain one or more aromatic rings, making them more polarizable because of the conjugated π systems in their structure. This fraction may also include sulfur-containing compounds such as thiophene and dibenzothiophenes due to their polarity being close to polyaromatic hydrocarbons, as well as their relatively lower molecular mass. Resins and asphaltenes, the more polar fractions, contain aromatic rings with heteroatom substituents such as nitrogen, sulfur, and oxygen. Resins are miscible in solvents such as heptane or pentane, whereas asphaltenes are defined by their insolubility in these solvents, as they precipitate in excess *n*-heptane or *n*-pentane. Asphaltenes consist of polycondensed aromatic rings with aliphatic side chains, which contribute to their solid nature. Distinguishing between these SARA fractions via advanced analytical methods such as gas chromatography and mass spectrometry is essential for understanding petroleum behavior and optimizing its industrial applications.

**Figure 1:**
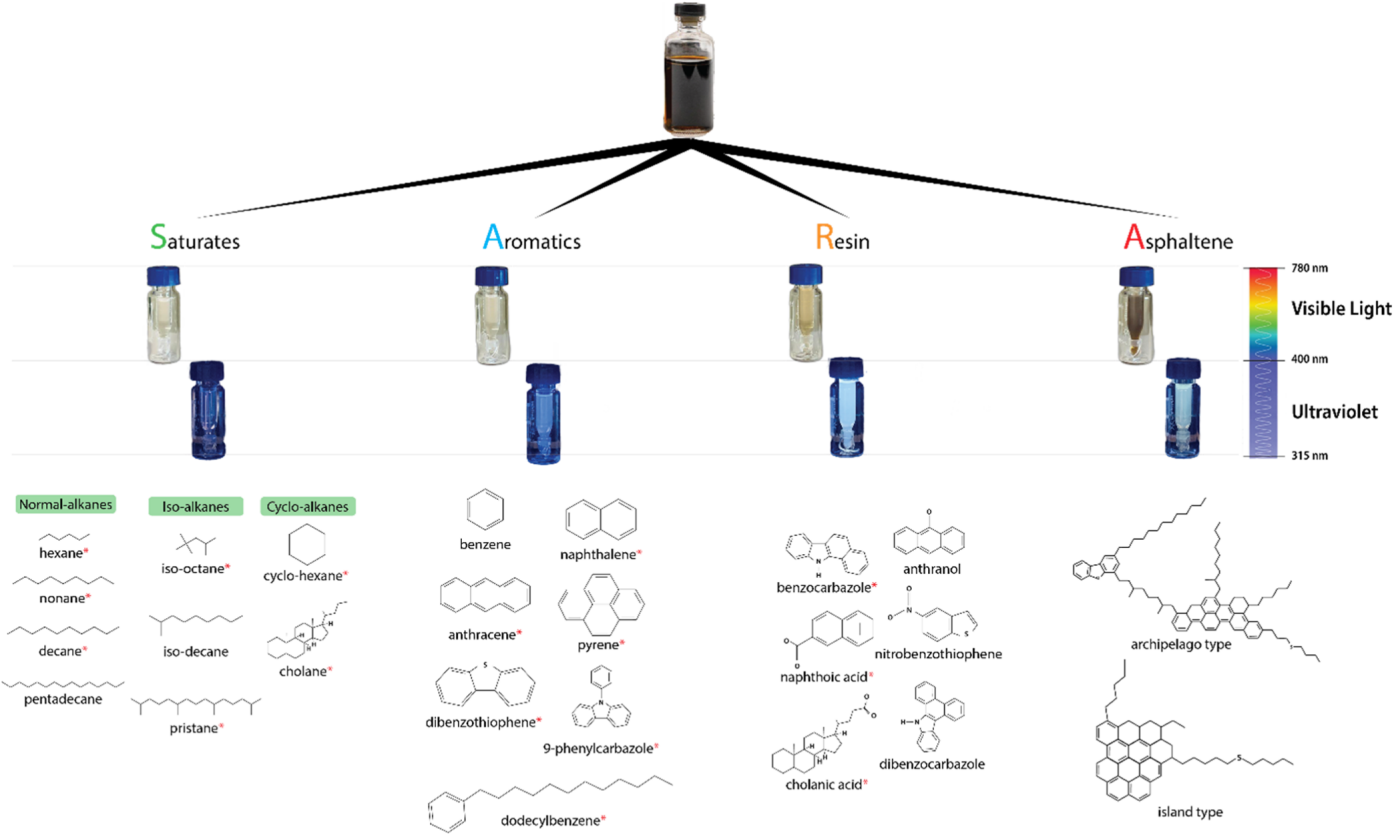
Visual representation of SARA (saturates, aromatics, resins, and asphaltenes) fractions. The vials display each fraction, with ambient light on the upper row and UV light on the bottom. Saturates appear clear with no fluorescence, whereas aromatics exhibit a slight yellowish hue under ambient light and fluorescence due to the presence of aromatic rings. Resins and asphaltenes show intense coloration and fluorescence under UV light, reflecting their complex aromatic structures and the presence of heteroatoms. The molecular structures of the key compounds are illustrated below each fraction. The modeled asphaltene structures are based on the work of Headen et al.^6^ The * symbols represent the model compounds used in the μSARA-HPLC method.

Petroleum SARA analysis is critical in guiding operations across the entire petroleum value chain, from upstream to downstream. In upstream operations, SARA analysis is essential for understanding crude oil composition, which directly influences reservoir management by enabling accurate modeling of fluid behavior under varying temperature and pressure conditions.^7–100^ This compositional analysis helps predict the stability of components such as asphaltenes and paraffins, which can precipitate and cause flowline blockages or damage production equipment.^10,11^ During transportation and storage, the mixing of incompatible fluids can lead to asphaltene self-aggregation, resulting in deposition and flow disruptions. SARA analysis plays a crucial role in anticipating such issues and ensuring fluid compatibility.^12,13^ In downstream refining processes, the distribution of saturates, aromatics, resins, and asphaltenes is pivotal for optimizing operations such as cracking and coking, as well as preventing fouling in equipment and deactivation of catalysts.^14–16^ By providing insights into the stability and behavior of different fractions, SARA analysis helps ensure efficient petroleum extraction, transportation, and refining, making it an indispensable tool for the industry.

The primary mechanism for separating SARA fractions relies on normal-phase chromatography, in which components are separated through adsorptive interactions between stationary and mobile phases. By gradually increasing the polarity of the mobile phase, different fractions can be eluted, while asphaltenes, which are insoluble, are precipitated and filtered out.^17–20^ Despite its utility, SARA methodologies often yield inconsistent results, making comparisons across different techniques unreliable. Variations in recovery rates, volatile losses, and solvent usage can lead to erroneous conclusions.^21–23^

Although modern automated high-performance liquid chromatography (HPLC) methods have improved reproducibility, they remain limited by long analysis times exceeding 1 h, the need for specialized configurations, and limited commercial accessibility.^24–27^ The objective of this study was to develop μSARA-HPLC, an optimized, highly reproducible HPLC-based SARA method requiring only small microvolume samples. This method reduces analysis time to just 35 min, including analysis and column regeneration, while maintaining precision and consistency. Additionally, μSARA-HPLC is implemented using commercially available components and an optimized solvent gradient, providing a practical and efficient solution for SARA analysis without requiring specialized instrumentation.

## History of SARA Method Development

The first documented SARA analysis method was introduced by Jewell et al. in 1972,^5^ and since then, numerous approaches have been developed to address the complexity of separating petroleum fractions. Given the intricate and highly variable nature of crude oil, SARA analysis has become an essential tool for understanding petroleum composition, behavior, and processing implications. Over the years, various techniques have emerged, with each aimed at balancing accuracy, reproducibility, and efficiency. For this work, we classify SARA methodologies into two broad categories: non-HPLC and HPLC-based approaches. Non-HPLC methods include the early techniques of open-column chromatography, which laid the foundation of the field, along with more recent advances. In contrast, high-performance liquid chromatography (HPLC)-based methods have gained prominence owing to automation and precision; nonetheless, they present unique challenges. By discussing these approaches, we illustrate the evolution of SARA analysis, highlighting how HPLC-based methods differ, the key limitations they present, and their potential for standardization in automated workflows. This classification highlights the increasing importance of HPLC as a preferred method for petroleum analysis, particularly due to its reproducibility and efficiency.

## Non-HPLC SARA Methods

Non-HPLC methods for SARA analysis often start with the deasphalting of petroleum samples using solvents such as *n*-pentane or heptane to separate the asphaltene fraction. The remaining maltene fraction, containing saturates, aromatics, and resins, is then passed through chromatography columns. In early techniques, bonded silica or unbonded amino stationary phases were employed, with different solvents selectively eluting the various fractions. By adjusting the polarity of the solvents, the saturates, aromatics, and resins were separated sequentially and quantified. The choice of stationary phase material, such as Al_2_O_3_ or silica, and its particle size significantly impacts separation efficiency, influencing adsorption behavior and fractionation selectivity. After separation, the excess solvents were evaporated, and individual fractions were measured gravimetrically to determine the SARA composition. Although these methods have been extensively documented,^4,5,19^ they suffer from significant drawbacks, for example, they are labor- and time-intensive. Additionally, achieving consistent and reproducible results across different laboratories remains a challenge with these methods, limiting their reliability for standardized analysis.^22,27–29^

Among non-HPLC methods, thin-layer chromatography with flame ionization detection (TLC-FID) has emerged as a promising and relatively fast approach for SARA analysis. Commercially available Iatroscan instrument have been widely adopted because of its ability to separate and detect the main hydrocarbon groups.^30,31^ However, reproducibility issues with FID responses for resins and asphaltenes remain problematic.^21^ This inconsistency is particularly significant for heavy crude oils and rock extracts, in which accurate quantification of these fractions is crucial for understanding sample composition and behavior.^30,32^ Additionally, TLC-FID is an inherently destructive technique; each peak is assumed to represent a single compound class, with no opportunity for further verification or cross-examination of the separated fractions.^27^ This limitation can lead to oversimplifications, especially in complex mixtures for which more detailed analysis would be beneficial.

In addition to chromatographic techniques, several nonchromatographic methods have shown potential for SARA analysis, although they remain largely in the research phase. Techniques such as nuclear magnetic resonance (NMR) spectroscopy, infrared spectroscopy, and microfluidics offer alternative approaches to fractionating and analyzing petroleum samples.^33–35^ Indirect optical measurements, such as attenuated total reflectance Fourier transform infrared spectroscopy (ATR-FTIR), provide rapid, nondestructive analysis but have not yet been validated for routine use in SARA analysis.^36^ While these emerging methods show promise for achieving faster, more efficient analyses, challenges with reproducibility and sensitivity remain, preventing their widespread adoption. Consequently, these methods are currently used to complement rather than replace established chromatographic approaches.

## HPLC Methods for SARA Analysis

The first use of HPLC for SARA analysis was reported by Suatoni and Swab in 1975,^37^ and the method involved a proprietary system developed by Gulf Research. This method required the deasphalting of samples prior to injection, the separation of the maltene fraction into saturates and aromatics while leaving resins in the column, and recalculating the fractions on the basis of mass balance. A subsequent method reported by Galya and Suatoni^38^ also depended on manually packed columns and deasphalting for pretreatment. While these early methods demonstrated the potential for using HPLC in SARA analysis, they were labor intensive and inconsistent due to variability in column packing and solvent compositional differences. In 1986, Grizzle and Sablotny^39^ refined the approach by introducing dual detectors and fraction collection verified by gas chromatography–mass spectrometry (GC–MS); however, this method still required deasphalting and had long runtimes.

Later,^21^ researchers developed an HPLC-based SARA method, but it also required deasphalting before analysis. These semiautomated methods suffered from variability in sample preparation, particularly in the deasphalting step, making reproducibility across laboratories difficult. The lack of standardized column packing and solvent selection and the long analysis times further hindered consistent implementation.

With recent advances in HPLC methods for SARA analysis, the need for deasphalting has been eliminated, simplifying analysis. In 2013, Boysen and Schabron^40^ introduced a PTFE column method that allows asphaltenes to precipitate directly within the system, a process they called SAR-AD (asphaltene determinator).^40^ While promising, this method involves manually packed columns and runtimes of up to 4 h, limiting broader application. Bissada et al.^27^ developed a dual-column system with automated backflushing and solvent polarity switching for gravimetric SARA determination without prior deasphalting. However, the method still faced reproducibility challenges, largely due to the use of proprietary column designs, and required approximately 1 h per sample to complete the analysis. Karevan et al.^24^ introduced a fully automated system with three commercial columns packed with PTFE, silica, and cyano materials; propane gas was used to enhance peak separation; however, the setup was complex and had runtimes of up to 3 h. Subsequent studies introduced optimizations such as adjusting sample concentration and selecting appropriate detectors to enhance signal detection. Additionally, reports explored the use of ethane injection to sweep the columns and improve peak separation efficiency.^25,26^ However, challenges with cross-laboratory reproducibility, custom PTFE column preparation, and secondary verification of separated fractions, along with issues related to detector sensitivity and solvent compatibility, continue to limit the widespread adoption of these methods.

## Methodology

### Samples and Materials

SARA analysis via HPLC was conducted on seven samples, including crude oil, gas condensate, and rock-extracted bitumen (Table 1). One sample served as a quality control reference: the North Sea Oil (NSO-1) standard from the Oseberg field, recognized by the Norwegian Petroleum Directorate (NPD). Additionally, five samples from various fields were analyzed, representing a range of API gravities from 10 to 50° (Table 1). The rock-extracted bitumen originated from immature, organic-rich Qusaibah shales (Silurian in age), with a total organic carbon (TOC) content of approximately 12% (Table 1). Bitumen was extracted via a Dionex 350 accelerated rock extractor with dichloromethane as the solvent. Because the bitumen sample is a solvent-diluted extract of solid rock organics, it does not reflect a native fluid and therefore is not suitable for accurate API gravity or density measurements.

**Table 1:** Crude Oil and Bitumen Samples Used in the μSARA-HPLC Analysis

sample ID	fluid type	source rock age	Density kg/m^3^	API gravity
NSO-1:	crude oil	Kimmeridgian	861	32.8°
APO-1	crude oil	Jurassic	997.2	10.4°
APO-2	crude oil	Cretaceous/Jurassic	925.2	21.5°
APO-3	crude oil	Jurassic	879.5	29.4°
RAO-1	crude oil	Jurassic	824.9	40.1°
APO-4	crude oil	Silurian	779.5	50.0°
RE-QHS	bitumen	Silurian	NA	NA

To ensure quality control, the NSO-1 standard was injected at the beginning and end of each run to monitor column performance and detect potential issues impacting separation stability. This approach ensured consistent results, as any deviation from the expected NSO-1 response signaled potential column fouling, solvent quality issues, or equipment malfunctions. Over four months, reproducibility was confirmed with NSO-1, with more than 100 crude oil injections performed for SARA analysis. This robust quality control ensured data accuracy and reliability, enabling prompt identification and resolution of inconsistencies.

All solvents used were of HPLC grade: *n*-pentane (CAS: 109–66–0), dichloromethane (CAS: 75–09–2), isopropyl alcohol (CAS: 67–63–0), and methanol (CAS: 67–56–1). Pure compounds were used to confirm the elution windows for each SARA fraction. The reference compounds were selected based on their structural resemblance to saturate, aromatic, and resin hydrocarbons commonly present in petroleum. These compounds were sourced from Chiron and Sigma-Aldrich to ensure high purity and reliability as analytical standards. For saturates, the compounds included hexane (CAS: 110–54–3), nonane (CAS: 111–84–2), decane (CAS: 124–18–5), isooctane (CAS: 540–84–1), pristane (CAS: 1921–70–6), cyclohexane (CAS: 110–82–7), and cholane (CAS: 548–98–1). For aromatics, dodecylbenzene (CAS: 123–01–3), naphthalene (CAS: 91–20–3), anthracene (CAS: 120–12–7), pyrene (CAS: 129–00–0), dibenzothiophene (CAS: 132–65–0), and 9-phenylcarbazole (CAS: 1150–62–5) were used. The reference compounds for the resins included benzocarbazole (CAS: 239–01–0), naphthoic acid (CAS: 93–09–4), and cholanic acid (CAS: 546–18–9). Asphaltenes were precipitated from a 10° API extra heavy crude sample, washed with heptane following the procedures outlined in ASTM D6560, and filtered through multiple cycles for use as model compounds.

## HPLC Instrument

The HPLC system for SARA fractionation utilized in this study included an Agilent 1260 Infinity II high-performance liquid chromatography system, which is designed for reliable and reproducible analysis of complex petroleum samples. This integrated system includes a series of interconnected components for efficient separation and precise quantification, all of which are controlled via the Agilent OpenLab Chromatography Data System. An overview of the system’s setup and key components is presented in Figure 2. Notably, all these components are commercially available from most HPLC manufacturers, allowing for the replication of this setup by other researchers interested in standardizing SARA analysis.

**Figure 2:**
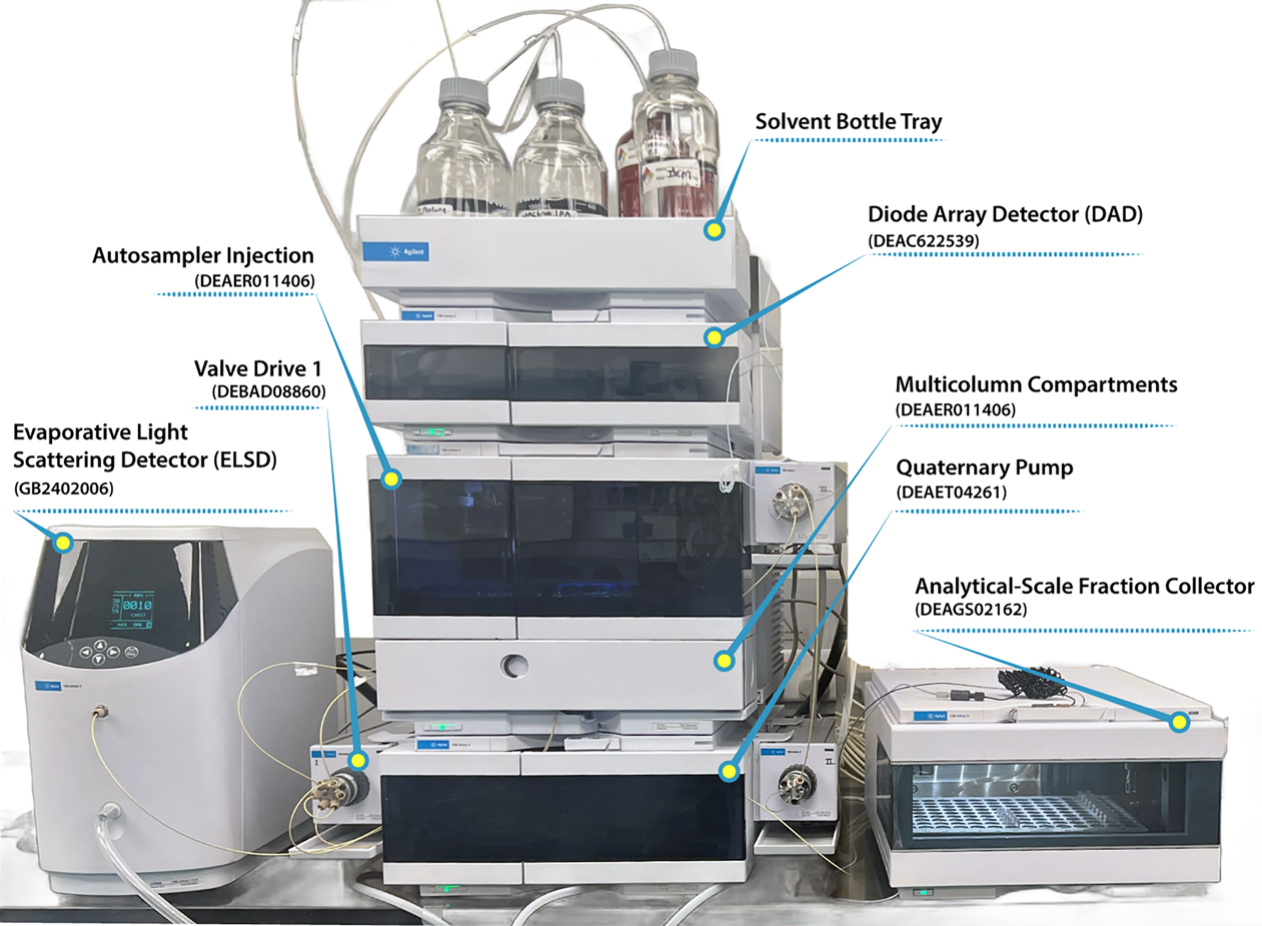
HPLC system used for μSARA-HPLC analysis. The system includes commercially available components, such as the Agilent 1260 Infinity II system, along with a Vialsampler, the ZORBAX RX-SIL and SB-CN columns, a Polaris Si-SA MetaGuard column, and dual detectors (ELSD and DAD).

The key components include the 1260 Infinity II Vialsampler, which accommodates pressures up to 600 bar and supports a broad injection range with metering devices of 0.1 and 100 μL, allowing for precise sample handling. Sample injections are directed through an advanced column configuration, starting with a Polaris Si-SA MetaGuard guard column (Part No. A2004MG2) placed first to isolate asphaltenes through an in-line filtration mechanism. The mobile phase, consisting primarily of *n*-pentane, facilitates separation based on component solubility characteristics. This is followed by a ZORBAX SB-CN column (Part No. 880975–905) optimized for resin fractionation and a ZORBAX RX-SIL column (Part No. 880975–901) for aromatic separations.

Detection is achieved through a dual-detector system incorporating both an evaporative light scattering detector (ELSD) and a diode array detector (DAD). ELSD offers high-level sensitivity for saturate fractions, particularly in gradient elution, whereas DAD is sensitive for detecting aromatic and polar compounds because of its wavelength-specific capabilities. DAD detection is performed at wavelengths of 254 nm for aromatics and resins and 350 nm for asphaltenes. After evaluating several wavelengths—including 254, 300, 350, and 400 nm—we selected 254 nm for aromatics and resins, as it is a well-established wavelength for aromatic detection.^21,41^ For asphaltenes, 350 nm was chosen based on our optimization studies, which indicated that this wavelength provides enhanced sensitivity for asphaltene content and is less influenced by changes in solvent gradient, unlike 254 nm during the asphaltene elution window. An analytical fraction collector fitted with low-dispersion tubing completes the setup, enabling the targeted collection of fractions for further analysis. The layout of these components, as illustrated in Figure 3, ensures robust and streamlined SARA analysis, with a focus on maximizing reproducibility and precision across various sample types.

**Figure 3:**
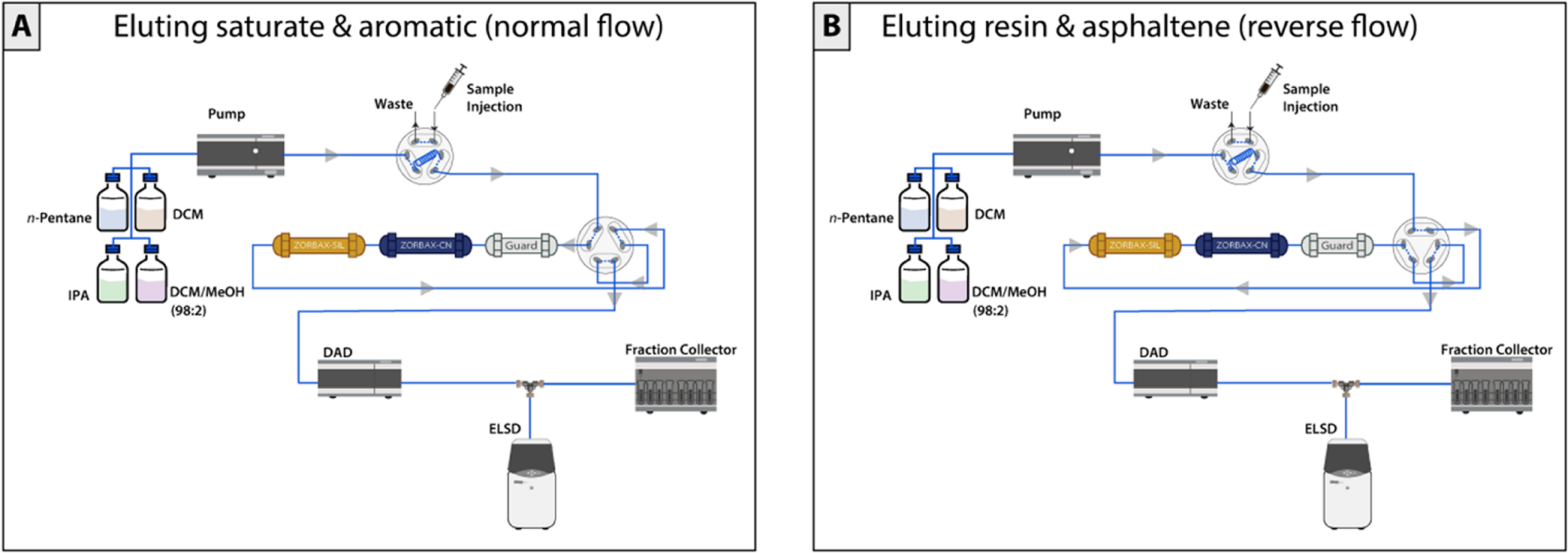
Schematic of the μSARA-HPLC system for fraction elution. (A) In the normal flow configuration, saturates and aromatics are eluted using *n*-pentane, with detection by ELSD and DAD. (B) In the reverse flow configuration, resins and asphaltenes are eluted by increasing solvent polarity with isopropyl alcohol (IPA), dichloromethane (DCM), and methanol (MeOH). After asphaltene elution, column washing and regeneration are performed on the system to restore *n*-pentane for the next run. Automated fraction collection enables confirmation of separation purity through secondary analytical methods.

In this method, crude oil samples are initially diluted with DCM at a 1:100 ratio by mixing 10 mg of oil with 100 μL of DCM. The diluted sample is then drawn into a syringe equipped with a Whatman UNIFLO 25 syringe filter, featuring a poly­(ether sulfone) membrane with a pore size of 0.22 μm. After filtration, the samples are transferred into 2 mL HPLC vials. For the HPLC run, an injection volume of 1.5–3 μL of the diluted sample is used, introducing approximately 0.15 to 0.3 mg of oil into the system. Injection volumes typically do not exceed 1.5 μL for heavy crude oils and bitumens with high resin and asphaltene content to ensure consistent column washing. A 3 μL injection is occasionally used for light and superlight crude oils (API > 46°) to improve detection, as these samples are easier to wash due to their low resin and asphaltene content. This small injection volume extends the column lifespan by minimizing residue buildup, reducing the need for maintenance and preventing unnecessary loading of oil. This approach not only optimizes the efficiency and reliability of each analysis but also helps maintain consistent separations across runs.

The HPLC system operates at a flow rate of 1.5 mL/min, and a preset solvent gradient program is utilized, which is detailed in Table 2, with *n*-pentane as the primary mobile phase. The method begins with forward flow for the initial 12 min, during which saturate and aromatic fractions are sequentially eluted. The 12 min elution window was optimized based on multiple test runs and pure injections of model compounds, along with confirmation by fraction collection and GC-MS analysis, ensuring complete separation of saturates and aromatics with no overlap. The ELSD detector first identifies and quantifies the saturate fraction, which elutes between 3 and 4 min, followed by the aromatic fraction, which is detected by the DAD, which elutes between 4 and 6 min (Figure 4). During this phase, asphaltenes are retained within the Polaris Si-SA MetaGuard column, whereas resins remain in the ZORBAX SB-CN column.

**Figure 4:**
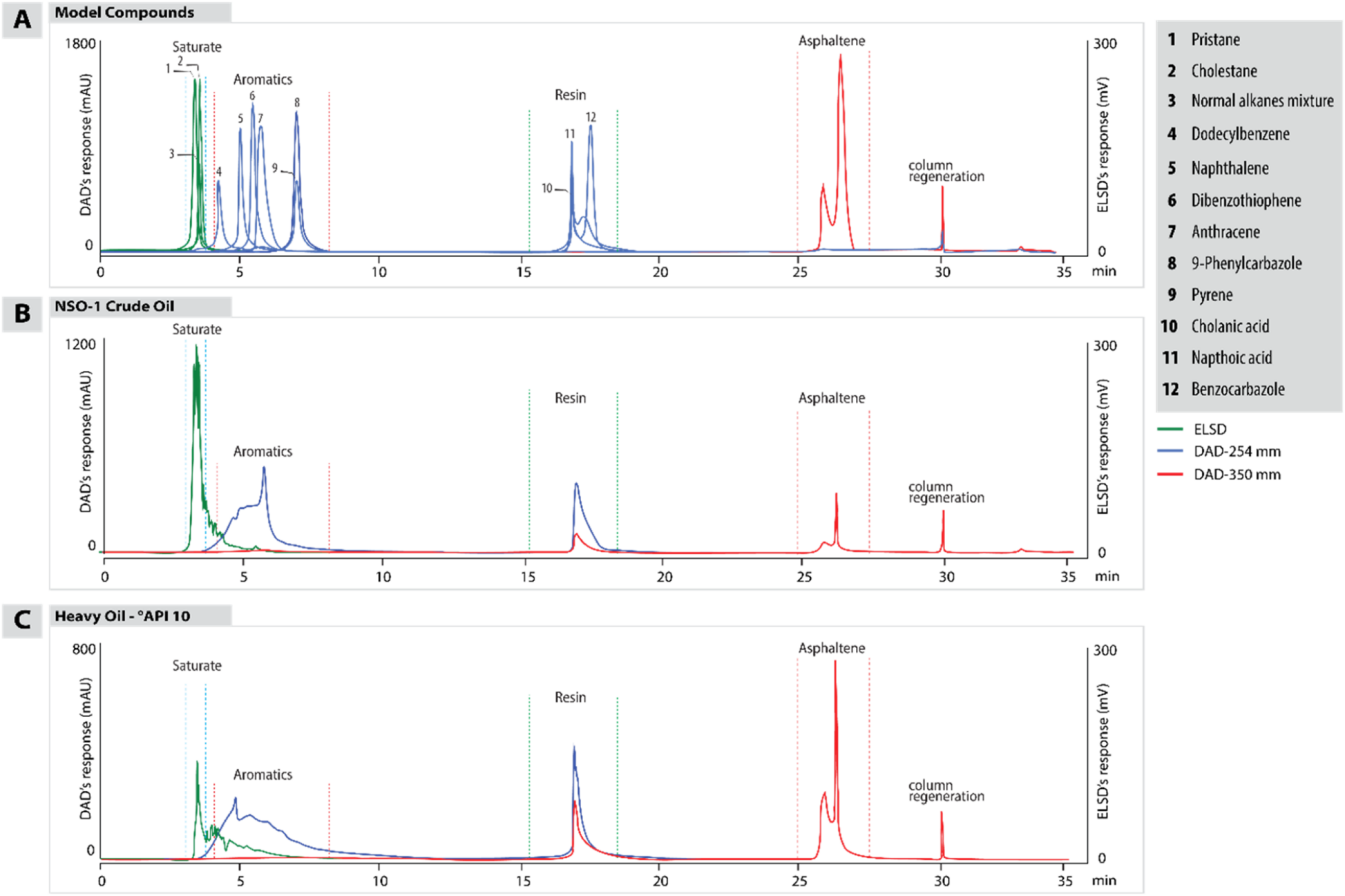
HPLC chromatograms with signals from ELSD and DAD at wavelengths of 254 nm for aromatics and resins and 350 nm for asphaltenes. (A) A composite chromatogram combining single injections of individual model compounds displaying their respective elution orders and clearly showing the sequential separation of saturates, aromatics, resins, and asphaltenes. (B) A chromatogram that represents NSO-1 crude oil, with notable peaks for high saturate and aromatic content. (C) A chromatogram that shows heavy crude oil (CO-1) with relatively high proportions of asphaltenes and resins, highlighting the suitability of the method for a wide range of oil types.

**Table 2:** Solvent Gradient and Run Program Steps^a^

step	time (min.)	solvents				flow (mL/min)
		% DCM	% *n*-pentane	% IPA	% DCM/MeOH (98:2)	
1	0	0	100	0	0	1.5
	12	0	100	0	0	1.5
2	12.5	12	88	0	0	2
	20	12	88	0	0	2
3	21	75	0	25	0	1.5
	27	0	0	45	55	1.5
4	28	0	0	25	75	1.5
	30	100	0	0	0	1.5
	30.5	0	100	0	0	2
	35	0	100	0	0	2

a 1: normal flow saturates and aromatics elution, 2: reverse flow resin elution, 3: reverse flow asphaltene elution, 4: column clean up and regeneration, DCM: dichloromethane, IPA: isopropyl alcohol, MeOH: methanol.

At the 12 min mark, the solvent flow is reversed to backflush the system, aiding in the elution of the resin and asphaltene fractions. To facilitate resin elution, the solvent polarity is increased by introducing a mixture of 12% DCM and 88% *n*-pentane, allowing the resin fraction to elute between 12.5 and 20 min. Further polarity adjustments are made for asphaltene elution: at 21 min, a 75:25 DCM to IPA mixture is introduced, followed by a 45:55 DCM/MeOH mixture at 24 min, with subsequent adjustments. Asphaltenes typically elute between 21 and 27 min. To prepare for the next sample, the system undergoes column cleanup with a 25:75 DCM to MeOH mixture at 26–28 min, followed by a forward flush with 100% *n*-pentane at an increased flow rate of 2 mL/min at the 30.5 min mark to ensure column regeneration (Table 2). This streamlined procedure allows for a total runtime of 35 min per sample, enhancing both efficiency and throughput. The solvent gradient program, detailed in Table 2, was optimized using model compounds to ensure efficient separation. During optimization, no asphaltenes were detected in the resin fraction, and vice versa, confirming that the assigned elution windows effectively isolate each fraction.

The method’s efficient runtime and precise solvent utilization also support sustainability by reducing solvent consumption. In each run, approximately 9.1 mL of Solvent A (DCM), 41.2 mL of Solvent B (*n*-pentane), 3.9 mL of Solvent C (IPA), and 6.5 mL of Solvent D (2% MeOH/98% DCM mixture) are consumed. This, along with the shorter analysis time, reduces solvent usage, minimizes waste and aligns with environmentally friendly laboratory practices.

For fraction collection, a time-based method is used to activate the fraction collector according to the elution order: saturates (3–4 min), aromatics (4–6 min), resins (12.5–20 min), and asphaltenes (21–27 min). Within the resin elution window shown in Table 2, the fraction collection criteria were set to collect the peaks between 16 and 17 min. Calibration curves are generated using the NSO-1 reference material, linking mass-based dilutions in parts per million (ppm) with detector responses—ELSD for saturates and DAD for the remaining fractions. The data from the HPLC chromatograms are processed via Agilent OpenLab CDS software, allowing for accurate analysis and quantification of each SARA fraction.

## Verification of SARA Fractions by GC-MS and FT-ICR-MS

The saturate and aromatic fractions were analyzed using an Agilent 7890B GC coupled to a 5977MS detector with helium as the carrier gas at 1.2 mL/min. A DB-5MS capillary column (60 m × 0.32 mm, 0.25 μm film) was used to separate the compounds in the oven at 60 °C (1 min), which was ramped up at 3 °C/min to 310 °C (15 min hold time). The injector was operated in splitless mode. The inlet temperature was set at 310 °C, and the MS was in full scan mode to inspect for cross-contamination. In the GC-MS analysis, a solvent delay of 7 min was implemented to protect the mass spectrometer’s filament, activating the detector only after the solvent had eluted. The key ions included *m*/*z* 57 for alkanes and *m*/*z* 91 for alkylbenzenes, along with aromatic markers such as methyl naphthalene (*m*/*z* 142), phenanthrene (*m*/*z* 178), dibenzothiophene (*m*/*z* 184), and triaromatic steroids (*m*/*z* 231).

Fourier transform ion cyclotron resonance mass spectrometry (FT-ICR MS) was performed with a 12T SolariX mass spectrometer (Bruker Daltonics) with atmospheric pressure photoionization (APPI) and electrospray ionization (ESI) sources in positive mode. The resin and asphaltene fractions in toluene were split between APPI (aromatic) and ESI (polar) ionization techniques. Each spectrum, collected over *m*/*z* 180–1800 with 50 scans and 8 million data points, was processed with a signal-to-noise threshold of 3 and validated via isotopic fine structure analysis.

## Results

### Optimized HPLC Separation of SARA

To validate the μSARA-HPLC method developed in this study, we first aimed to optimize the separation of SARA components by using model compounds to define precise elution windows. The model compounds used to define elution windows are described in detail in the Methodology section under Samples and Materials. This foundational step allowed for refinement of the solvent gradients, detector parameters, and chromatographic conditions to achieve robust and reproducible separation. The HPLC chromatograms in Figure 4 display results from individually injected model compounds (Figure 4A) and two crude oil samples: NSO-1 oil (reference material, Figure 4B) and a heavy crude oil with an API gravity of 10 (CO-1, Figure 4C). Chromatographic separation allows for the sequential elution of SARA fractions, clearly separating saturates, aromatics, resins, and asphaltenes, each with unique detector signals (Figure 4).

The analysis begins with the elution of the saturate fraction, in which alkanes, owing to their nonpolar nature, elute first with minimal interaction with the stationary phase. These compounds are followed by single-ring aromatic compounds such as dodecylbenzene, progressing to naphthalene, anthracene, and the four-ring compound pyrene. Each aromatic compound exhibits distinct, sequential elution, and the phase reversal timing is used to optimize solvent polarity and flow direction for subsequent fraction separations. Following the elution of aromatics, an increase in solvent polarity facilitated resin elution, whereas the final stage, involving the highest-polarity solvent mixture, allowed for asphaltene elution.

The crude oil chromatograms (Figure 4B,C) show similar elution patterns, with clear peaks for saturates and aromatics. However, the complexity of crude oil becomes evident in the aromatic, resin, and asphaltene fractions. Unlike model compounds, which display discrete peaks, crude oil aromatics have multiple maxima, indicating a mix of compounds with varying degrees of aromaticity. This complexity is especially pronounced in the heavy crude oil sample, where overlapping peaks suggest numerous multiring aromatic compounds (Figure 4C).

The peak intensities of the resin fraction varied, reflecting diverse molecular structures within the crude oil. Despite these complexities, chromatographic reproducibility was confirmed across multiple injections, with consistent peak patterns observed in each run. This consistency supports the reliability of the HPLC method for separating and identifying SARA fractions in complex petroleum samples.

Notably, the asphaltene fraction in the heavy crude oil sample (Figure 4C) exhibits two distinct peaks, which can be attributed to the presence of multiple asphaltene subfractions with varying molecular structures and polarities. In contrast, the NSO-1 oil sample (Figure 4B), characterized by a higher API gravity, shows a single asphaltene peak with a minor shoulder, indicating a less complex asphaltene composition. This variation in peak patterns aligns with the differences in asphaltene content and composition between heavy and light crude oils.

### Verification of SARA Fraction Purity and Composition via GC-MS and FT-ICR-MS analysis

GC–MS analysis was used to confirm the purity of the HPLC-separated saturate and aromatic fractions, as shown in Figure 5. Although ELSD and DAD signals are overlaid for illustration in Figure 4, each detector output is processed independently, with no cross-detection between fractions. This was validated by extracting time slices from the apparent overlapping region and analyzing them by GC-MS. The chromatograms displayed total ion counts and diagnostic ions specific to alkanes and aromatic compounds, with no cross-contamination detected between fractions. The absence of *m*/*z* 91 in the saturate fraction verifies that even the least polar aromatics, such as dodecylbenzene, were effectively separated, ensuring the retention of more polar aromatic compounds. Similarly, the absence of *m*/*z* 57 in the aromatic fraction confirms that no alkanes were present, validating the purity of the separation. The saturate fraction presented a typical *n*-alkane profile, allowing for the identification of hydrocarbons down to C8. For the aromatic fraction, BTEX compounds (benzene, toluene, ethylbenzene, xylenes), naphthalenes, and phenanthrenes were confirmed, particularly in the NSO-1 oil, supporting the reliability of the method in separating distinct, pure fractions.

**Figure 5:**
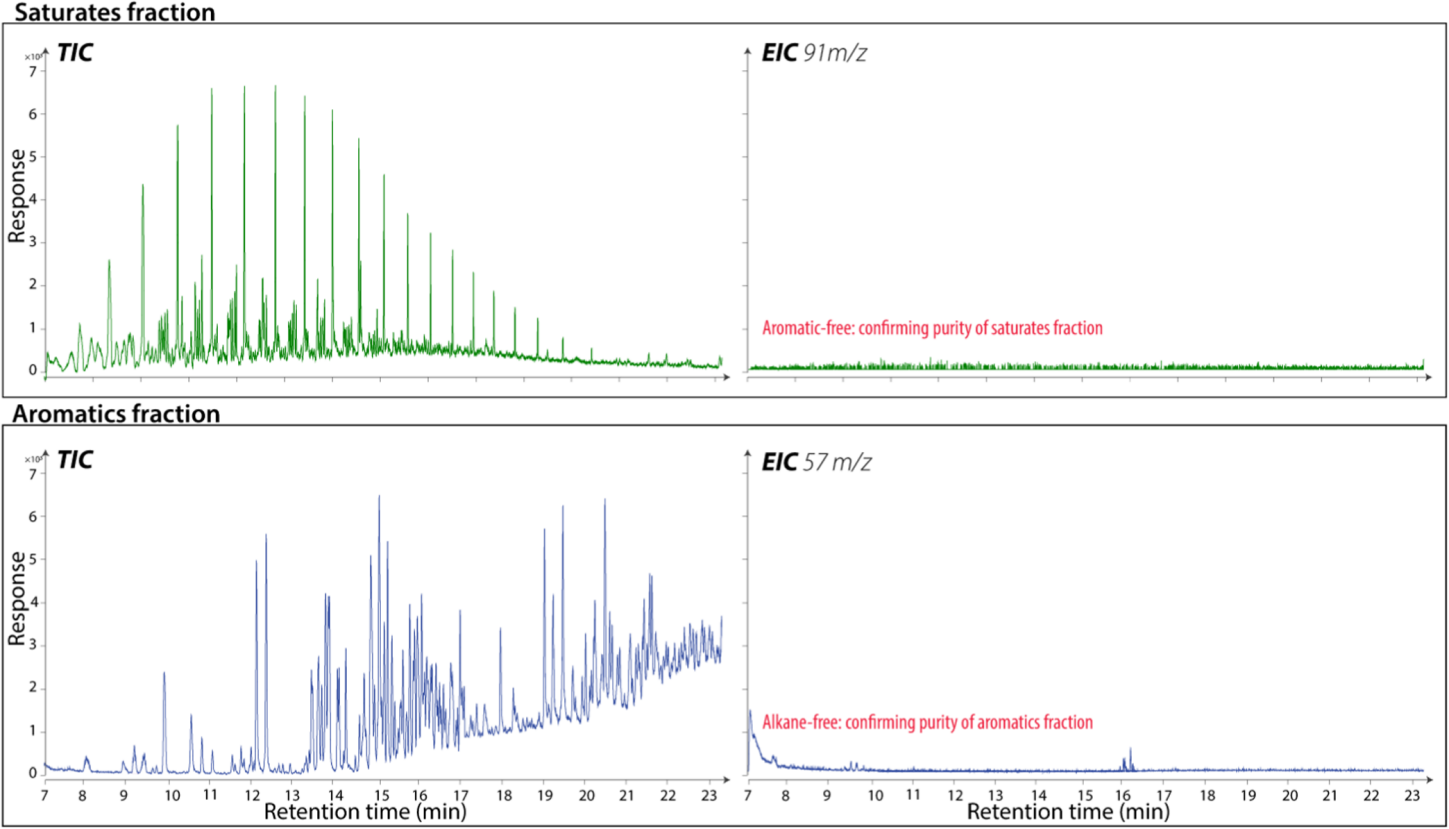
Secondary verification of the saturate and aromatic fractions through GC–MS chromatograms. The top panel displays the total ion chromatogram (TIC) for the saturate fraction, alongside the extracted ion chromatogram (EIC) at *m*/*z* 91 (a major mass fragment for aromatic rings with alkyl substituents). The bottom panel shows the TIC for the aromatic fraction, with the EIC at *m*/*z* 57 (a major mass fragment for alkanes), confirming that the aromatic fraction is free of alkanes.

FT-ICR-MS was employed for the resin and asphaltene fractions, which are not suitable for GC–MS analysis because of their relatively high polarity. Figure 6 displays van Krevelen diagrams showing hydrogen-to-carbon (H/C) versus oxygen-to-carbon (O/C) ratios, color-coded by double bond equivalents (DBEs) and circle sizes representing carbon numbers. The resin fraction exhibited a broader DBE range,^6–20^ indicative of lower aromaticity and smaller molecular sizes, with an assigned molecular formula (AMF) count of 6689. In contrast, the asphaltene fraction displayed a DBE range of 11–50, reflecting higher aromaticity and complexity, peaking near C_80_, with an AMF count of 13,148—almost double that of the resin fraction. Pie charts confirmed CHONS as the predominant heteroatom class in both fractions, highlighting their complex composition and the distinct characteristics of each component. These analyses collectively validate the capability of the method to achieve high-purity separation of SARA fractions, providing robust molecular-level insights into each component.

**Figure 6:**
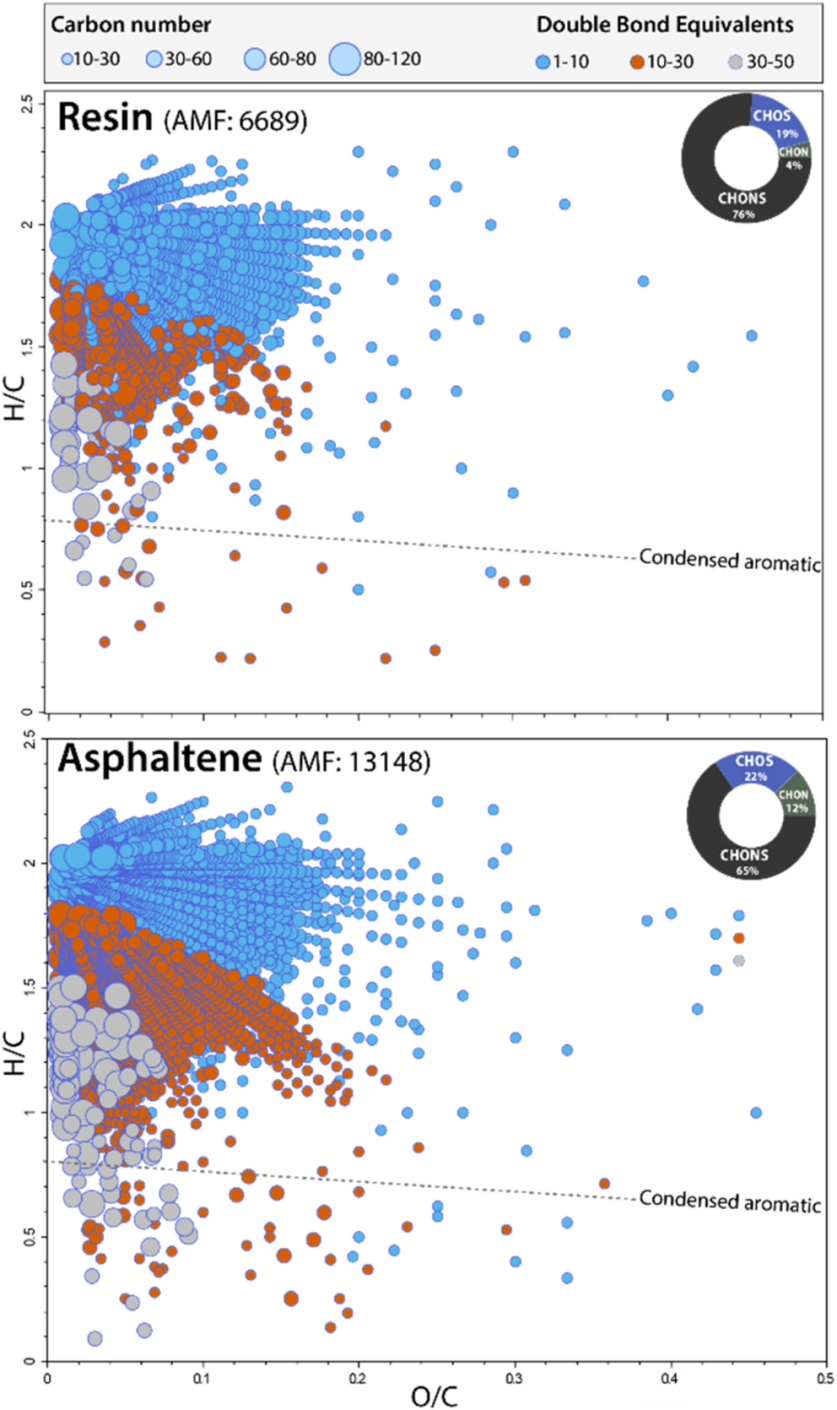
Secondary verification of the resin and asphaltene fractions via FT-ICR-MS analysis. The van Krevelen diagrams display hydrogen-to-carbon (H/C) versus oxygen-to-carbon (O/C) ratios for both fractions. The color code represents different double bond equivalents (DBEs), while the circle size corresponds to the number of carbon atoms. AMF stands for assigned molecular formula.

### Calibration, Linearity, and Reproducibility

To ensure the reliability of the μSARA-HPLC method for routine SARA analysis, we validated its reproducibility, accuracy, and quantification capabilities through detector linearity tests, calibration curve generation, and long-term reproducibility studies. These experiments aimed to demonstrate the robustness and consistency of the μSARA-HPLC system across different sample types and experimental conditions.

Detector linearity was tested across a range of injection volumes. Figure 7 shows cross plots of NSO-1 crude oil injections (1–5 μL), demonstrating linear responses for both the ELSD and DAD detectors at 254 and 350 nm. The correlation coefficients (*R* ^2^) ranged from 0.96 to 0.99, indicating strong linearity between detector response and sample volume. This finding supports the method’s robustness, ensuring consistent SARA quantification over various concentrations.

**Figure 7:**
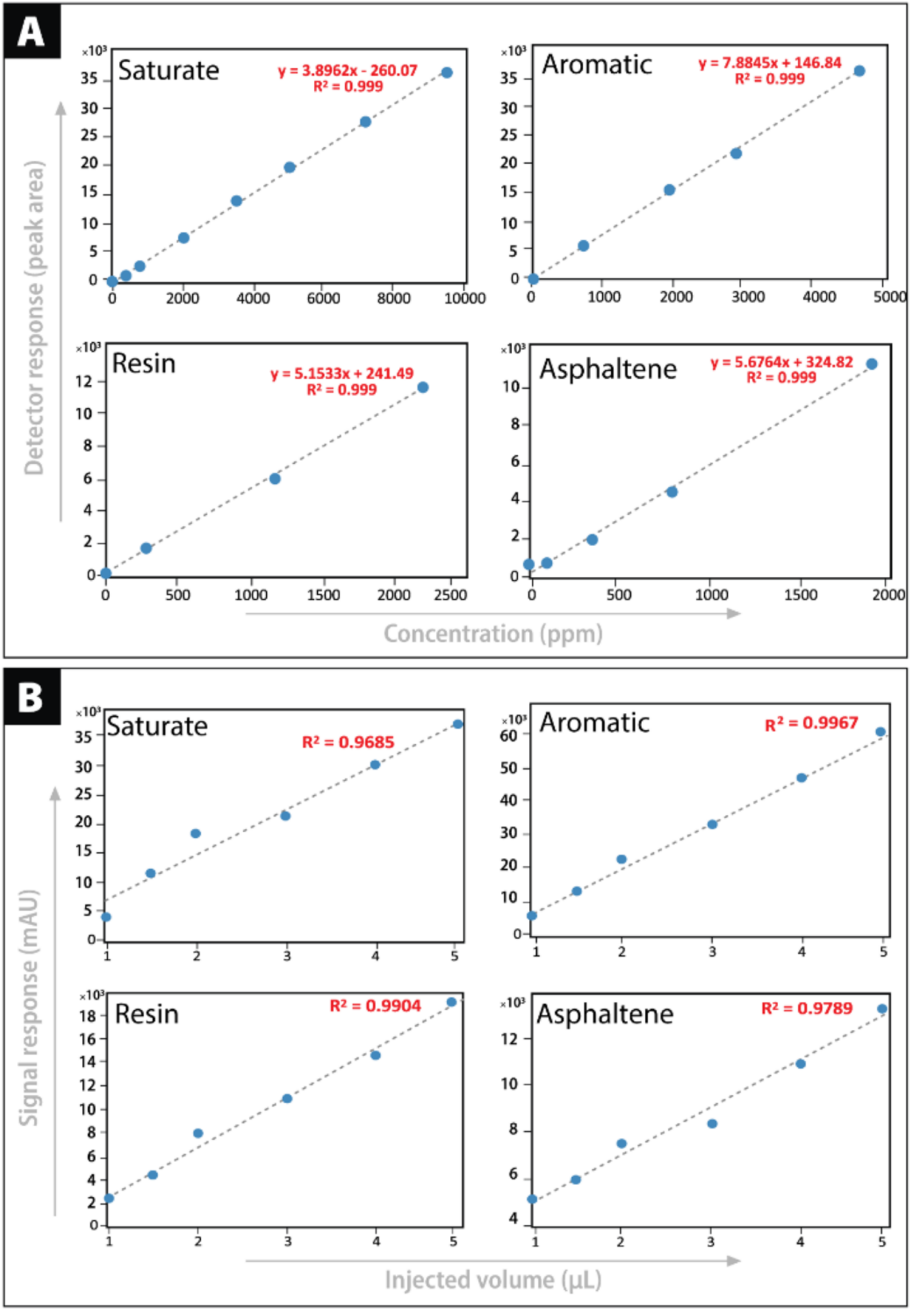
Calibration curve cross-plots showing the signal versus concentration for each SARA fraction. (A) Detector response plotted against concentration (ppm) for saturates, aromatics, resins, and asphaltenes; (B) Signal response plotted against injection volume (μL).

Calibration curves were also generated for the NSO-1 sample postfractionation. The mass of each fraction was measured after solvent evaporation, and dilutions were prepared to generate curves plotting their concentration (ppm) against detector response. Figure 7 indicates similar linearity (*R* ^2^ = 0.99) for all fractions. These calibration equations enabled accurate quantification of compounds according to mass and percentage composition, reinforcing the method’s precision.

The reproducibility of the μSARA-HPLC method was evaluated over three months using crude oil samples with API gravities ranging from 10 to 50, the NSO-1 reference oil, and a bitumen extract. As shown in Figure 8, the relative standard deviation (RSD%) for all the examined samples remained below 1%, confirming robust repeatability. In addition, the method’s reliability has been benchmarked against conventional SARA analysis techniques, with a separate manuscript dedicated to addressing cross-contamination issues associated with ASTM D2007, a well-documented drawback noted in prior studies.^22,27^ Together, these results demonstrate that μSARA-HPLC offers high accuracy, precision, and reproducibility, making it a reliable tool for SARA analysis in diverse petroleum samples.

**Figure 8:**
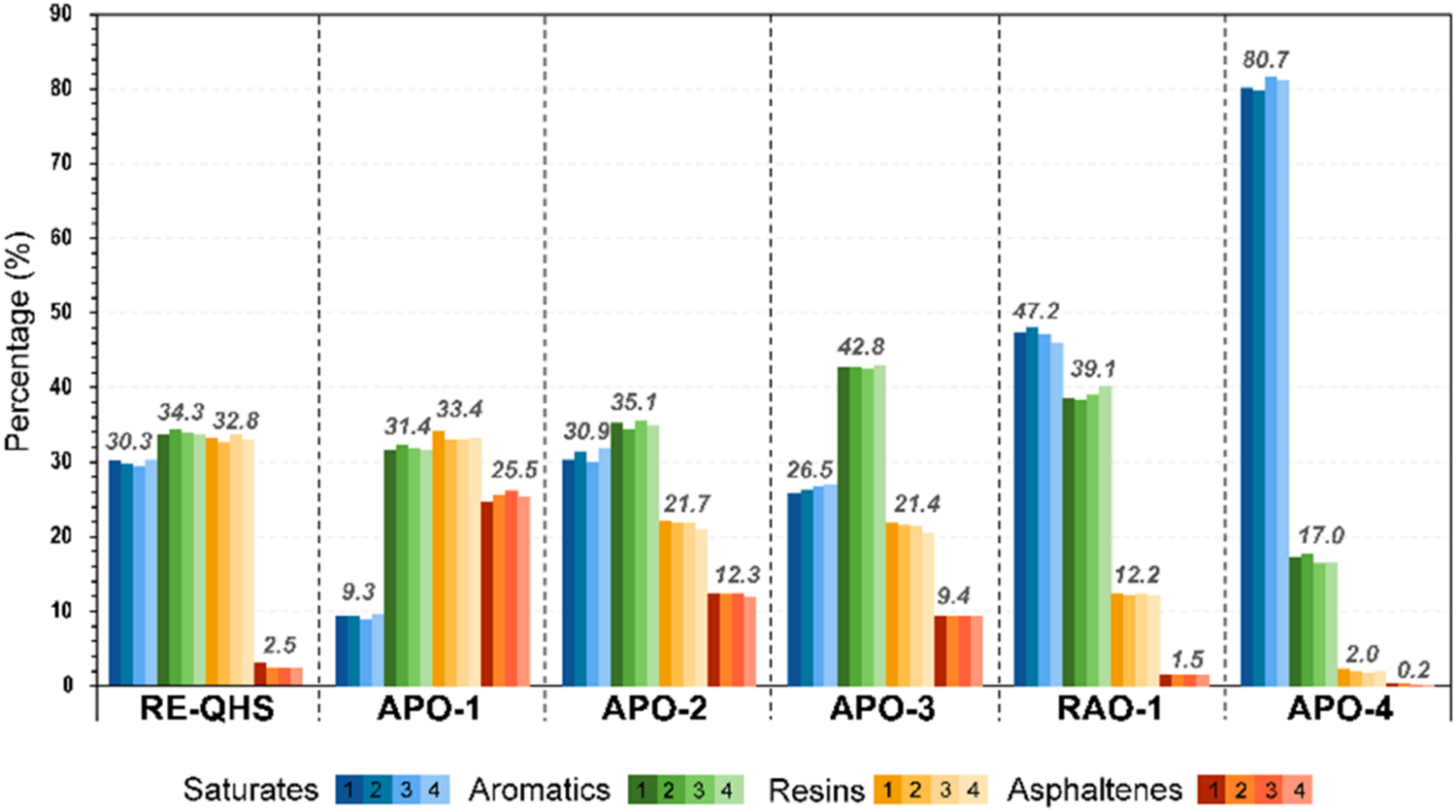
Percent composition of SARA fractions (saturates, aromatics, resins, and asphaltenes) for crude oils with different API gravities and bitumen extracts. Each sample was analyzed four times, with standard deviation (SD) values below 1% for all fractions.

### NSO-1 as Validation Benchmark

The NSO-1 reference oil is a well-known standard, analyzed by numerous laboratories globally and well-documented in several studies.^42–46^ The SARA composition of NSO-1 has been reported in The Norwegian Industry Guide to Organic Geochemical Analyses.^47^ As shown in Table 3, our laboratory has conducted multiple runs to validate the consistency and accuracy of our results; comparing them to the accepted ranges reported.^47^ The methods utilized to document these ranges include analyzing the deasphaltened NSO oil sample using TLC-FID and liquid chromatographic separation. The consistency of our analysis and similarity to the reference bulk composition results confirm the reproducibility and reliability of our measurements.

**Table 3:** Repeatability Analysis of NSO-1 Sample^a^

sample	SAT	ARO	RESIN	ASPH
(name)	(%)	(%)	(%)	(%)
NSO-1_1	54.084	28.994	12.143	4.779
NSO-1_2	53.558	29.685	12.408	4.349
NSO-1_3	53.511	29.93	12.526	4.032
NSO-1_4	54.613	28.721	12.832	3.834
NSO-1_5	54.06	29.733	12.469	3.738
average	53.965	29.413	12.476	4.146
NSO-REF-TLC-FID	40–60	25–42	5–13	1–4
NSO-REF-LC	56–58	26–30	13–19	1–4

a NSO-REF refers to accepted ranges from The Norwegian Industry Guide to Organic Geochemical Analyses.^47^

## Discussion

### Overcoming HPLC-SARA Method Challenges

HPLC-based SARA methods have historically faced significant challenges related to reproducibility, runtime, and system complexity. Many existing approaches rely on custom configurations involving manually packed columns, components from different manufacturers, or highly specialized systems that are difficult to replicate across laboratories.^21,24–27,40^ These factors, coupled with lengthy analysis times—often exceeding several hours—hinder standardization and the routine adoption of such methods.^24–26^ Our study addresses these limitations by developing an optimized HPLC SARA method using a fully integrated, commercially available system from Agilent. Since off-the-shelf hardware, software, and data acquisition components are used, the method can be easily replicated by researchers without complex customizations. Moreover, the method reduces the runtime to just 35 min per sample, making it a practical solution for both research and industrial applications. In this work, we aim to establish a reproducible, accessible, and efficient approach to SARA analysis, contributing to a more standardized practice in petroleum characterization.

### Enhanced Detection and Injection Efficiency

Addressing the intertwined challenges of reproducibility and sensitivity discussed earlier, the integration of dual detectors (ELSD and DAD) in our method allows for minimal sample injection. For example, injecting 1 μL of oil diluted at 10 mg in 100 μL of DCM introduces only 0.1 mg of oil into the HPLC, a significantly lower mass than other HPLC-SARA methods, which often require larger volumes and masses.^21,27,40^ This low injection volume reduces sample consumption, extends the column lifespan by minimizing residue buildup, and enhances overall cost efficiency while maintaining reliable results with minimal waste.

Previous studies, such as those by McLean and Kilpatrick,^48^ highlighted significant challenges with asphaltene adsorption onto silica columns, especially with large sample injections. Their methods, which involved up to 8–10 g of pretreated crude oil adsorbed onto 50 g of silica gel, led to irreversible binding of polar compounds, including asphaltenes. In contrast, in our method, only 1.5 μL of a highly diluted crude oil sample is used, approximately 1/5000th of the volume/mass used by McLean and Kilpatrick.^48^ This reduced injection volume significantly decreases the risk of asphaltene adsorption, ensuring clean separation and effective recovery of SARA fractions. Additionally, our use of backflushing, similar to the method of Bissada et al.,^27^ has been shown to be effective in preventing asphaltene precipitation. Bissada et al.’s work demonstrated that routine backflushing could maintain column integrity over multiple runs without significant asphaltene fouling. In our study, after more than 200 runs, the system pressure remained stable between 50–120 bar, indicating no asphaltene buildup or irreversible adsorption. In contrast, a steady increase in pressure would indicate adsorption issues. These results underscore the importance of low injection volumes, appropriate dilutions, and regular backflushing for maintaining method robustness and reproducibility over extended use.

### Standardization and Future Directions

The complexity of crude oil, particularly in the resin and asphaltene fractions, continues to pose challenges for SARA analysis. While saturate and aromatic fraction characterization is well established, our understanding of the complex resin and asphaltene fractions remains insufficient. Although models have been proposed to describe these fractions, their inherent complexity prevents any method from fully capturing their properties.^49^ With incremental advances in SARA methodologies, new complexities are often introduced, making it difficult for researchers to replicate results or adopt methods across different laboratories.

A significant limitation in current practices is the absence of standardized reference materials for cross-laboratory comparisons. Most studies rely on unique sample sets, with limited efforts toward coordinated analyses across multiple laboratories. This lack of standardization makes it difficult to validate methods and ensure consistency. Although some researchers share samples for blind analysis, this ad hoc practice is far from ideal.

To advance SARA analysis, we propose the use of standardized reference oils, such as those in our study, to benchmark and cross-validate methods. We are committed to sharing these reference materials to provide a consistent point of comparison for laboratories. A shared standard would promote consistent, reliable SARA analysis, enabling better characterization of the most complex components in petroleum. This step toward standardization could bridge the gap between methodological improvements and practical applications in research and industry.

## Conclusions

In this study, we introduce an optimized and reproducible HPLC-based SARA analysis method that effectively addresses the limitations of existing techniques by reducing system complexity and analysis time. By utilizing a commercially available, fully integrated system, we achieve comprehensive separations within 35 min while maintaining high precision and reproducibility across diverse petroleum samples. The method incorporates sensitive detectors, such as ELSD and DAD, to facilitate accurate analysis of saturates, aromatics, resins, and asphaltenes, requiring minimal sample injection volumes and low solvent usage. This method provides a standardized and efficient approach with strong potential for widespread adoption in petroleum analysis, enhancing consistency across laboratories and advancing the field of SARA analysis.

## Supplementary Material



## Notes

SARA fraction percentages of measured crude oil and bitumen samples, giving quadruplicate values and RSD for each fraction (PDF)

## Abbreviations

μSARA-HPLC: micro SARA high-performance liquid chromatography
SARA: saturates, aromatics, resins, and asphaltenes
HPLC: high-performance liquid chromatography
ELSD: evaporative light scattering detector
DAD: diode array detector
TLC-FID: thin-layer chromatography with flame ionization detection
GC-MS: gas chromatography–mass spectrometry
FT-ICR-MS: Fourier-Transform ion cyclotron resonance mass spectrometry
API: American Petroleum Institute (gravity measurement)
DBE: double bond equivalent
AMF: assigned molecular formula
BTEX: benzene, toluene, ethylbenzene, xylenes
NSO-1: North Sea Oil Standard
APO: Arabian platform oil
RAO: Rub’ alKhali oil
