# Supplementary material for: Accelerating Saturate, Aromatic, Resin, Asphaltene (SARA) Analysis for High-Fidelity Petroleum Profiling via μSARA-HPLC
Source: ACS Omega. 2025 Jun 23;10(26):27806–18. doi: 10.1021/acsomega.5c00439 (PMC12242619; doi:10.1021/acsomega.5c00439)
Supplement: Supplementary file 1 [file ao5c00439_si_001.pdf]

This document is confidential and is proprietary to the American Chemical Society and its authors. Do not copy or disclose without written permission. If you have received this item in error, notify the sender and delete all copies.

Accelerating Saturate, Aromatic, Resin, Asphaltene (SARA) Analysis for High-Fidelity Petroleum Profiling via μSARA-HPLC

Journal:	*ACS Omega*
Manuscript ID	ao-2025-00439z.R2
Manuscript Type:	Article
Date Submitted by the Author:	15-Apr-2025
Complete List of Authors:	Atwah, Ibrahim; Saudi Aramco,<br>Alsaif, Maram; Saudi Aramco, EXPEC ARC<br>Usman, Muhammad; King Abdullah University of Science and Technology<br>Abu Alreesh, Mohammed; The University of Texas at Austin Jackson School of Geosciences

SCHOLARONE™
Manuscripts

Sample ID	Saturates %	Aromatics %	Resin %	Asphaltene %
RE-QHS	30.70	33.33	33.14	2.83
	29.86	35.20	32.48	2.46
	29.81	34.77	33.24	2.19
	30.81	34.08	32.69	2.42
RSD%	1.52	2.06	0.95	9.38
APO-1	9.47	31.53	34.19	24.81
	9.29	32.20	32.97	25.54
	8.84	31.87	33.12	26.17
	9.54	31.73	33.30	25.43
RSD%	2.94	0.77	1.42	1.89
APO-2	30.38	35.23	22.04	12.35
	31.37	34.46	21.89	12.28
	30.02	35.65	21.88	12.44
	31.94	35.01	21.06	12.00
RSD%	2.48	1.22	1.77	1.34
APO-3	25.86	42.83	21.95	9.35
	26.37	42.74	21.56	9.33
	26.69	42.56	21.31	9.43
	27.08	42.90	20.56	9.46
RSD%	1.69	0.30	2.38	0.58
RAO-1	47.44	38.58	12.44	1.55
	47.98	38.42	12.12	1.48
	47.15	39.13	12.28	1.44
	46.08	40.30	12.10	1.53
RSD%	1.47	1.88	1.12	2.87
APO-4	80.13	17.19	2.32	0.37
	79.86	17.82	2.00	0.32
	81.72	16.46	1.71	0.10
	81.26	16.57	2.05	0.12
RSD%	0.95	3.20	10.72	52.32
NSO-1	54.05	28.99	12.14	4.78
	53.56	29.69	12.41	4.35
	53.51	29.93	12.53	4.03
	54.61	28.72	12.83	3.83
	54.06	29.73	12.47	3.74
RSD%	0.74	1.59	1.77	9.14
